# Health-Related Quality of Life in Male Patients under Treatment for Substance Use Disorders with and without Major Depressive Disorder: Influence in Clinical Course at One-Year Follow-Up

**DOI:** 10.3390/jcm9103110

**Published:** 2020-09-26

**Authors:** Julia E. Marquez-Arrico, José Francisco Navarro, Ana Adan

**Affiliations:** 1 Department of Clinical Psychology and Psychobiology, School of Psychology, University of Barcelona, Passeig de la Vall d’Hebrón 171, 08035 Barcelona, Spain; jmarquez@ub.edu; 2 Department of Psychobiology, School of Psychology, University of Málaga, Campus de Teatinos s/n, 29071 Málaga, Spain; navahuma@uma.es; 3 Institute of Neurosciences, University of Barcelona, 08035 Barcelona, Spain

**Keywords:** health-related quality of life, substance use disorder, dual disorders, major depressive disorder, dual depression, relapses

## Abstract

Health-related quality of life (HRQoL) assessment has interest as an indicator of degree of affectation and prognosis in mental disorders. HRQoL is impaired in both Substance Use Disorder (SUD) and Major Depressive Disorder (MDD), two conditions highly prevalent, although less studied when both are coexisting (SUD + MDD). Hence, we decided to explore HRQoL with the SF-36 survey in a sample of 123 SUD and 114 SUD + MDD patients (51 symptomatic and 63 asymptomatic of depressive symptoms) under treatment. We performed analyses to examine HRQoL among groups, and its predictive value at 3-, 6- and 12-month follow-ups through regression models. Patients with SUD + MDD had worse HRQoL than SUD patients and population norms. For Mental Health, Vitality, and General Health dimensions, lower scores were observed for SUD + MDD regardless the presence/absence of depressive symptoms. For Physical Functioning and Health Change, depressive symptomatology and not the comorbidity of SUD + MDD diagnoses explained HRQoL limitations. At 3-, 6- and 12-month follow-ups we observed two predictors of relapses, General Health for asymptomatic SUD + MDD, and Physical Functioning for SUD. Improving HRQoL in SUD + MDD may be targeted during patient’s treatment; future studies should explore the influence of HRQoL on patient’s prognosis taking into account the presence/absence of depressive symptomatology.

## 1. Introduction

Health-related quality of life (HRQoL) is one of the constructs with more interest in recent years as an indicator of treatment results in patients with different mental disorders. These include substance use disorder (SUD), severe mental disorders, and comorbidity among them, known as dual disorders [1,2,3]. Both from the field of research and from clinical practice, different indicators of success of the treatment or recovery of the patient are currently being investigated, beyond the simple reduction of psychiatric symptoms and withdrawal from substance use [4,5]. There are different published studies that explain the relevance of certain variables that function as indicators of the patient’s prognosis [6,7,8], among which is quality of life [8,9]. Undoubtedly, having indicators at the start of treatment that report a poor clinical evolution can be key for adjusting the intervention to the specific needs of each patient [10,11].

The HRQoL is a multidimensional construct of special relevance since it reports the effects that the disorder has on the patient’s daily functioning, the degree of involvement generated by the disorder, and the subjective perception of the limitations that they experience in their daily life [12,13]. In this way, the HRQoL study provides important guidance for establishing treatment goals and knowing the degree of functional recovery of the patient beyond the reduction of symptoms, which is always a goal for therapeutic intervention [3,14].

Several studies indicate that patients with dual disorders have a worse HRQoL compared to patients with only one diagnosis [12,15,16]. Comorbidity between an SUD and a mental disorder has been consistently associated with worse quality of life in different domains. However, most of the studies carried out to date are cross-sectional and they have been developed with reduced sample sizes of dual disorder patients without considering the possible effect of the symptomatology from the comorbid mental disorder. It has been found that patients with dual disorders exhibit worse physical functioning and vitality [12,17]; thus, they experience, for example, daily limitations in their physical activities and they tend to be worn-out every day. Dual disorder has been also linked to worse mental health, in some cases with symptoms such as depression, anxiety, nervousness, and frequent insomnia [14,17,18] and to greater limitations in social functioning [14,18,19] as compared with patients who are only diagnosed with SUD. For example, patients may demonstrate problems when they have to attend to social meetings or family reunions due to their health. Therefore, previous studies point out clearly that dual disorders are linked to several limitations that affect patients’ daily cognitive and social functioning; however, no study has assessed the specific limitations considering psychiatric diagnosis as a differentiating variable.

If the most prevalent diagnoses of severe mental disorder in patients with dual disorders are considered, it is found that major depressive disorder (MDD) is one of the most frequently diagnosed conditions [19,20,21,22]. MDD is also associated with a worse quality of life [23,24,25]. MDD patients show worse HRQoL compared to the normal population, highlighting their low scores in the domains of social functioning [25,26,27], vitality, mental health, and emotional role [27,28,29]. Both the nature of the affective symptoms of MDD and the limitations generated by the disorders, at the cognitive and social levels, are consistently linked to a loss of quality of life [29].

More specifically, if the presence of a comorbid SUD and MDD is analyzed, a special severity is seen in this type of patient. This coexistence of diagnoses is related to more frequent depressive episodes [30] and of greater severity [31], a high risk of suicide [32], a worse prognosis in relation to addiction [33], and significant problems at the socioeconomic level [34]. For all these reasons, the study of the characteristics of patients with dual depression (SUD + MDD) and their HRQoL is of special interest, since it can provide valuable data on their adherence to treatment, prognosis and/or recovery [3,9,35,36]. The exploration of the influence of SUD vs. SUD + MDD on HRQoL is also of great interest due to its theoretical and clinical implications, since it could provide data on which HRQoL dimensions are especially affected by each of these conditions.

One of the most widely used instruments in mental health to study HRQoL is the Ware-Sherbourne SF-36 survey [37]. The SF-36 reports dimensional data for eight primary scales: Physical Functioning, Role-Physical, Role-Emotional, Social Functioning, Mental Health, General Health, Bodily Pain, and Vitality. It also provides a self-perceived item measuring changes in general health over the last year (Health Change item). This instrument has provided significant data when evaluating HRQoL in patients with SUD [38,39,40], MDD [13,41], as well as with dual pathology [5,42]. It is an instrument that can be used as an indicator of the evolution of the patient with SUD and, in addition, it has been shown to be sensitive to the presence of the diagnosis of MDD, having even suggested that it may be useful as a screening instrument [26,43].

Despite the data regarding HRQoL, which have revealed a negative impact on SUD, MDD, and comorbidity (SUD + MDD), very few studies have analyzed this topic. Most of the studies published to date have focused on the presence of SUD or MDD comorbidly to an organic pathology, but very few studies have used HRQoL in patients with SUD and MDD as the main diagnostic condition, or comorbidity between them. Furthermore, none of those studies have assessed the predictive role of HRQoL throughout the course of patient’s treatment for either SUD or MDD. The study of patients with SUD vs. SUD + MDD can extend the knowledge about dual depression and its specific therapeutic needs, as well as informing about possible targeted treatment that consider the patient’s subjective perspective and their insights. All these data could provide a subjective complementary perspective to clinicians, which also results in information that could be used to enhance the effectiveness of health care.

Furthermore, these studies use cross-sectional designs and only describe the dimensions of HRQoL without exploring its possible predictive role throughout the course of patient treatment. In the present study, we propose to analyze the differences in HRQoL in a sample of patients with SUD compared to patients with SUD + MDD (we expect to observe poorer HRQoL in patients with SUD + MDD than in SUD). Moreover, we intend to elucidate the possible predictive value of HRQoL at 3, 6, and 12 months of follow-up. In addition, we will explore differences and possible relationships based on the presence/absence of depressive symptoms in patients with SUD and MDD in order to identify their contribution both in the dimensions of quality of life and in the evolution during one year of follow-up. This study aims to provide data that encourage the adoption of an integrated recovery-oriented model that considers wider outcomes than abstinence as the main goal of treatment [1,15,18].

## 2. Experimental Section

### 2.1. Participants

A total of 237 voluntary patients were recruited and assigned to two groups according to their clinical diagnoses: SUD (N = 123) and SUD + MDD (N = 114; dual disorder condition). This study was a multicenter research, with participants from different public and private clinical centers specialized in SUD and mental health with inpatient and outpatient programs. Among these centers there were therapeutic communities, addiction treatment units, hospital’s mental health units, and community addictions centers, which are mostly addressed to men. The inclusion criteria followed for this study were: (1) male gender (based on the greater prevalence of men in SUD and dual disorder diagnoses, and also due to their larger presence in our clinical centers); (2) aged 19 to 55 years; (3) a current diagnosis of SUD, including those with addiction in early remission according to DSM-5 criteria (abstinence period from 3 to 12 months, without relapses in the last 3 months); (4) a diagnosis of MDD for the dual disorder condition established according to DSM-5 criteria [44]; (5) currently under treatment for their MDD, but in a clinically stable condition. All patients in our sample were in treatment programs for their corresponding diagnoses and had obtained negative results in all their abstinence urine analyses. Patients within the SUD group did not have history of previous MDD. On the other hand, the exclusion criteria followed were: (1) Meeting DSM-5 criteria for a current substance-induced disorder; (2) meeting DSM-5 criteria for any current diagnosis different than SUD or MDD; (3) psychiatric condition due to medical disease; (4) unstable or uncontrolled symptomatology (i.e., withdrawal); (5) inability to complete study interviews or instruments.

### 2.2. Procedure

In the first place, patients were screened in their respective treatment centers by their treating psychiatrist and/or psychologist who followed the inclusion/exclusion criteria. These patients passed through and extensive assessment and evaluation protocol at the beginning of their treatment (since they are part of a larger study) in each of their centers, which includes discarding substance-induced mood disorders and confirming their diagnosis (in our case the SUD and the MDD). Once the patients agreed to participate, they were provided and signed an informed consent. A post graduate psychologist from our research group assessed the patients individually and also collected the follow up data at 3, 6, and 12 months. For the follow-up a structured 21-item questionnaire was used, specifically designed for our study. The main variables registered during follow-ups were relapses (presence/absence), patients’ treatment status (“in treatment,” “drop-out” when the patient abandoned treatment against medical advice, and “discharged” when the patient left treatment because it has achieved therapeutic goals and professionals are advising treatment to finish), suicide attempts (yes/no), number of medical appointments attended, and need for medical assistance (yes/no). None of the participants were compensated for their participation and the only benefit they obtained was a report of their results. 

The assessment protocol was approved by the Research Committee of the University of Barcelona (IRB00003099) and is part of a wider research project. The present study complies with the tenets of the Declaration of Helsinki.

### 2.3. Measures

#### 2.3.1. Sociodemographic and Clinical Variables

We designed a structured interview specifically for our study to conduct with each patient in order to assess variables such as age, civil status, years of schooling, social situation, and living arrangements, among others. This interview was also employed to collect data on diagnosis according to DSM-5 criteria, personal/family psychiatric and medical records, history of suicide attempts, and SUD/MDD age of onset. In addition, the structured clinical interview for DSM-IV-TR Axis I Disorders (SCID-I) was administered to collect other clinical variables, such as medication prescribed, hospitalizations, type and number of drugs used, and abstinence period, as well as to confirm patients’ diagnoses. The SCID-I for DSM-IV-TR was used since the corresponding Spanish version for the DSM-5 was not available at the time of conducting the present study.

We used the drug abuse screening test (DAST-20) in its Spanish version [45] to obtain a measure of the SUD characteristics in the SUD and SUD + MDD groups. The DAST-20 provides a total severity score ranging from 0 to 20 (1–5 low; 6–10 intermediate; 11–15 substantial; 16–20 severe), with higher scores indicating that a more intensive therapeutic intervention was recommended. Regarding psychiatric symptoms, depressive symptoms in patients with SUD + MDD group were measured with the Spanish version of the 17-item Hamilton depression rating scale [46], its cut-off points being: 0–7, no current depression (asymptomatic condition); 8–13, low; 14–18, mild; 19–22, severe; and >23, very severe depressive symptoms. Following these criteria confirmed by clinical professionals’ opinion, patients in the SUD + MDD group were classified in subgroups as asymptomatic (Hamilton from 0 to 7) and symptomatic (Hamilton from 8 up to 23). Moreover, the patient’s motivation and social behavior were assessed, as an additional measure of the MDD, with the Social Adaption Self-evaluation Scale (SASS) [47]. The instrument consists of 21 items, with a range from 0 to 60, considering a score of less than 25 as social maladjustment, between 35 and 52 as normal, and those higher than 55 indicating an over-adaptation.

#### 2.3.2. Health-Related Quality of Life

For a measure of HRQoL we used the SF-36 survey in its Spanish version [48]. The SF-36 is a 36-item measure of HRQoL and consists of eight primary components: Physical Functioning (performing all physical daily activities from dressing and bathing to the most vigorous ones and its limitations due to health), Role Physical (reports the existence of problems in working and other daily activities due to health), Role Emotional (reports the existence of problems in working and other daily activities due to emotional problems), Social Functioning (measures the interference with normal social activities due to physical or emotional problems), Mental Health (reports the existence of nervousness and depression), General Health (evaluates personal health and the belief about how it is going to progress in the future), Bodily Pain (measures the existence of severe and extremely limiting pain), and Vitality (refers to the level of feeling tired and worn out all the time). Scores in the SF-36 range from 0 to 100, where a higher score indicates a better HRQoL and lesser limitations. This instrument includes an additional self-perceived item measuring changes in general health over the last year (Health Change item). The questionnaire also provides two secondary composite standardized scales using T scores (with a mean of 50 and standard deviation of 10): the Physical Health Component Summary and the Mental Health Component Summary. There are Spanish normative data for the scores of the primary scales of the SF-36 in the general population [48], which we will use to compare the groups of patients in our study.

#### 2.3.3. Statistical Analysis

Firstly, descriptive statistics (frequencies, means, and standard errors) were obtained for the sociodemographic and clinical variables for the SUD and SUD + MDD groups, as well as for the clinical characteristics of MDD in symptomatic and asymptomatic patients SUD + MDD. The possible differences in such variables among groups were explored by univariate analyses of variance (ANOVA) for continuous data. Nonparametric tests were conducted with Chi-Square statistic calculated according to the type of variable analyzed and the comparison groups.

For exploring HRQoL dimensions, we first performed multivariate analyses of covariance (MANCOVA) introducing the SF-36 dimensions as dependent variables, and the group (SUD and SUD + MDD) as the independent variable, in order to detect differences in HRQoL. We also considered treatment modality and the main substance of dependence as possible interaction factors for HRQoL in these variance analyses. A secondary MANCOVA was performed considering, in this case, the SUD group and two SUD + MDD subgroups depending on their symptomatic or asymptomatic condition. Thus, for this secondary MANCOVA, post-hoc comparisons were adjusted by Bonferroni’s correction so as to identify the role of depressive symptoms in HRQoL for the SUD, symptomatic SUD + MDD, and asymptomatic SUD + MDD groups. In all the variance analyses, age was considered as a covariate to control its possible effect, given that our groups differed significantly in this variable, with the SUD + MDD patients being older on average.

The predictive value of HRQoL at 3, 6, and 12 months of follow-ups was explored through logistic regression coefficients and linear regressions depending on the type of variable. Variables were dummy coded (1 = yes/0 = no) in the case of categorical variables such as presence of relapses, treatment status (in treatment, discharged, drop-out), and suicide attempts; the quantity of medical consultations attended was treated as a continuous variable. Logistic regression coefficients and their standard errors were back-transformed to generate odds-ratios (ORs) and their 95% confidence intervals. An attrition analysis was performed so as to explore the possible baseline differences among the participants who completed or abandoned our study for sociodemographic and clinical variables. All statistical analyses were carried out using the SPSS/PC+ statistics package (version 17.0, SPSS Inc., Chicago, IL, USA), and tests were two-tailed with the type I error set at 5%.

## 3. Results

### 3.1. Results in Sociodemographic and Clinical Variables

The participants in our sample had a mean age of 39.35 years (*SD* = 8.69), with patients with SUD + MDD being older than patients with SUD (*p* < 0.001). As we can see in Table 1, the patients in the sample were mostly single or divorced/separated, with no significant differences between groups in the marital status variable. Regarding the level of studies, the average years of schooling (10.95 years; *SD* = 2.64) places our sample below the level of secondary education in Spain, without significant differences according to diagnosis. On the other hand, regarding the economic situation, significant differences between groups were observed. While in the SUD + MDD group there is a high percentage of disability pension and, to a lesser extent, unemployment, in the group with SUD the conditions of being working or unemployed as well as without economic income are predominant (*p* < 0.001).

In relation to clinical characteristics, a greater presence of medical illness and medication use was observed in the SUD + MDD group compared to the SUD group (*p* < 0.001); antidepressants were the drugs most used by both groups. On the other hand, the history of suicide attempts and the number of them was higher in the group with SUD + MDD (*p* < 0.001).

Likewise, regarding the clinical characteristics of the SUD group, no differences were observed between groups in the substance of consumption, the most prevalent being alcohol, cocaine, and cannabis in both groups. Results on the DAST-20 scale indicate that both SUD + MDD and SUD patients have a substantial need for treatment (mean 12.92; SD = 4.09), without significant differences between groups. There were also no differences in the DAST-20 scale considering the presence/absence of depressive symptoms for patients in the SUD + MDD group. The mean of months of abstinence in the total sample was 8.73 months (SD = 3.40) without significant differences between diagnostic groups. There were also no differences between groups in the variable age of onset of SUD, nor in the years of its duration.

Regarding depressive psychiatric symptoms (see Table 2), the SUD + MDD group was subdivided according to the score on the Hamilton scale in asymptomatic (scores ≤ 7; N = 63; 44.7%) and symptomatic patients (scores ≥ 8; N = 51; 55.3%). The consideration of the presence of symptoms did not provide significant differences either in the age of onset of depression or in the years of its duration. Likewise, the data on the SASS scale of social adaptation did not show differences between asymptomatic and symptomatic SUD + MDD patients, and in both cases the scores were in the normal range.

### 3.2. Results in Health-Related Quality of Life for the SUD and SUD + MDD Groups

The analysis between groups considering the presence/absence of comorbid MDD (SUD + MDD vs. SUD groups) is presented in Table 3. Significant differences were observed in the dimensions of Physical Functioning (*p* = 0.036), Social Functioning (*p* = 0.022 ), Role Emotional (*p* = 0.012), Mental Health (*p* < 0.001), Vitality (*p* = 0.001), and General Health (*p* < 0.001), as well as for the Health Change item (*p* = 0.001). In all cases, the SUD + MDD group exhibited lower scores compared to the SUD group. Treatment modality (the highest contrast for the Vitality dimension: *F*_(2,235)_ = 1.795: *p* = 0.636; ηp^2^ = 0.005) and the main substance of dependence (the highest contrast for Mental Health dimension: *F*_(2,235)_ = 2.471; *p* = 0.520; ηp^2^ = 0.008) did not provide significant results either for the main effects for HRQoL or in the interaction between groups.

The analysis of the differences among the groups considering the presence/absence of depressive symptoms in the SUD + MDD group (symptomatic vs. asymptomatic subgroups, see Table 4), indicated that the lowest scores in the Physical Functioning and Health Change dimensions observed in the SUD + MDD group were explained by those symptomatic patients (*p* < 0.001 in all cases), since the scores of the asymptomatic SUD + MDD patients did not differ from those of the SUD group. On the other hand, in the Mental Health, Vitality, and General Health dimensions, the lowest scores of patients with SUD + MDD compared to the SUD group were observed regardless of the presence/absence of depressive symptoms (*p* < 0.001 in all cases). In this second analysis, the differences in the Social Functioning and Role Emotional dimensions obtained by not considering the depressive symptoms in SUD + MDD have disappeared.

Regarding the composite scales, the comparison between the SUD + MDD and SUD groups indicates differences only in the Mental Health Component scale (*p* < 0.001), with the SUD + MDD group having the lowest score (see Table 3). The difference in Mental Health Component is observed regardless of the presence/absence of depressive symptoms for patients in the SUD + MDD group (see Table 4).

### 3.3. Population Values in the SF-36 and Scores Obtained in the SUD and SUD + MDD Groups

**Figure 1:**
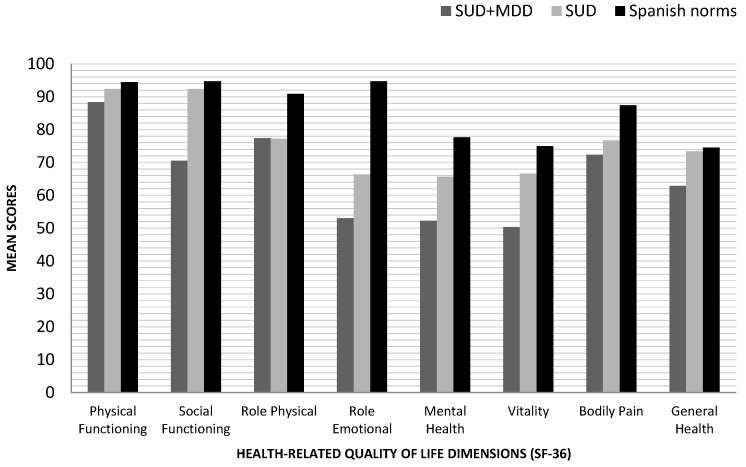
Mean scores in health-related quality of life dimensions (SF-36) according to the diagnosis, comparison with Spanish normative data from healthy individuals. SF-36: The Short Form Health Survey; SUD + MDD: Substance Use Disorder with comorbid Major Depression Disorder; SUD: Substance Use Disorder.

The comparison of the scores of our patients with respect to the population data (see Figure 1), allows us to add that both groups (SUD and SUD + MDD) present scores below the expected average in the dimensions of Role Physical, Role Emotional Mental Health, Vitality, and Bodily Pain of SF-36, although the impact is greater in the SUD + MDD group. Furthermore, both in the SUD group and in the SUD + MDD group, the values are similar to those of the general population in Physical Functioning. Considering the scales of Social Functioning and General Health of HRQoL, the SUD group shows adequate values, while in the case of the SUD + MDD group these are lower.

**Figure 2:**
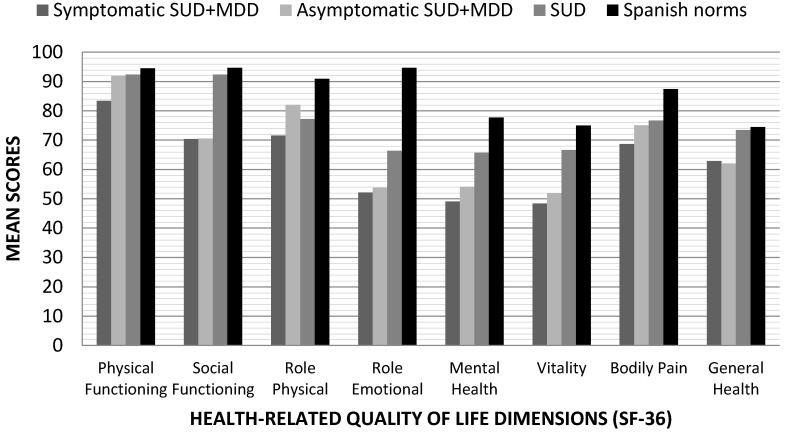
Mean scores in health-related quality of life dimensions (SF-36) according to the diagnosis and considering the presence of depressive symptomatology in dual depressed patients compared with the Spanish normative data in healthy individuals. SF-36: The Short Form Health Survey; SUD + MDD: Substance Use Disorder with comorbid Major Depression Disorder; SUD: Substance Use Disorder.

The HRQoL analysis, evaluating the presence/absence of depressive symptoms in the SUD + MDD group (see Figure 2), allows us to clarify that symptomatic patients obtain the lowest scores in Physical Functioning, Role Physical, Mental Health, Vitality, and Bodily Pain compared to population data. Finally, in the case of the Social Functioning dimension, SUD + MDD patients, both symptomatic and asymptomatic, show lower scores compared to normative data.

### 3.4. Predictive Value of Health-Related Quality of Life Dimensions at 3, 6, and 12 Months of Follow-Up

At 3 months of follow-up, we observed that some dimensions of the SF-36 have a predictive value for both the SUD and the asymptomatic SUD + MDD groups (see Table 5). The asymptomatic patients with SUD + MDD who were still on treatment were those with lower scores on Emotional Role (*p* = 0.008). The presence of relapses as early as three months is associated in SUD patients with a lower score in Physical Functioning (*p* = 0.009) and in asymptomatic patients with SUD + MDD with high scores in General Health dimension (*p* = 0.026). Furthermore, the score in the Vitality dimension was negatively linked to the number of medical consultations in asymptomatic SUD + MDD (*p* = 0.001) and with the need for medical care in the SUD group (*p* = 0.033). Finally, at three months of follow-up, no quality of life dimension provided significant relationships with the variables studied for symptomatic patients in the SUD + MDD group.

As shown in Table 5, at 6 months of follow-up we observed that high scores in the Bodily Pain dimension were related to having been discharged from the treatment in patients with SUD (*p* = 0.023); thus, the patients with the highest scores received the highest number of medical discharges. On the one hand, high scores in Vitality were related to being under treatment in symptomatic patients with SUD + MDD (*p* = 0.023), while lower scores were related to the need for medical care in SUD patients (*p* = 0.008). On the other hand, the lower the Physical Functioning score the more frequent was the presence of relapses in the SUD group (*p* = 0.034); and the lower the General Health score, the greater the number of medical visits required by patients in the asymptomatic group with SUD + MDD (*p* < 0.001).

Finally, at 12 months of follow-up (see Table 5), the Emotional Role dimension was associated with having been discharged from the treatment in patients with SUD (*p* = 0.025), while the high scores in the Vitality dimension were linked with continuing treatment in symptomatic patients with SUD + MDD (*p* = 0.015). Likewise, the lower the score in the Bodily Pain dimension, the more frequent was the dropping-out treatment for patients in the symptomatic SUD + MDD group (*p* = 0.012). On the other hand, low scores in Physical Functioning were linked to the presence of relapses in the SUD group (*p* = 0.038). In patients of the asymptomatic SUD + MDD group, high General Health scores were associated with the presence of relapses (*p* = 0.010) and with a lower need for medical consultations (*p* = 0.002).

In all follow-up analyses (3, 6, and 12 months), treatment modality and the main substance of dependence did not work as interaction factors for none of the groups (*p* > 0.752 for ORs and *p* > 0.358 for lineral regressions).

Regarding follow-up data, we observed that no subject was lost from our study at 3 and 6 months of follow-ups, but at 12 months follow-up 65 patients were missed (29 with SUD + MDD and 36 with SUD). We did not find any significant differences between those subjects who completed the study nor for those who were in treatment and those who drop-out/were discharged (*p* > 0.274) (see Table 6 and Table 7).

## 4. Discussion

In this study we aimed to analyze the differences in HRQoL between two groups with SUD considering the presence/absence of a MDD comorbid diagnosis, as well as its relationship with follow-up data during 12 months. Our main findings point out that patients with SUD + MDD have more limitations in their quality of life due to health than patients with SUD and no comorbidity. Therefore, dual depressed patients have poorer Physical Functioning, Social Functioning, Mental Health, and General Health; they also experience fewer positive health changes in the last 12 months (Health Change Item). When the presence of depressive symptoms was controlled for patients with SUD + MDD we observed that these were only relevant for the limitations in Physical Functioning and Health Change Item; depressive symptoms were not explaining the majority of the primary outcomes observed whereas the comorbidity of SUD + MDD itself did. The different dimensions of the SF-36 seem to have a greater predictive value for asymptomatic SUD + MDD and SUD patients than for symptomatic SUD + MDD patients.

The results obtained at the sociodemographic level indicate that our sample is similar to that described in previous studies carried out in patients with a dual disorder [15,17], with patients in the group with dual depression being mostly pensioners due to illness, while patients who did not show psychiatric comorbidity were either working or on sick leave due to the SUD. Regarding the clinical characteristics linked to the SUD, we highlight that the main substances of use are in line with the previous data [5,16], with alcohol, cocaine, and cannabis being the most prevalent in both groups. Furthermore, the age of onset of SUD in dual depressive patients was earlier than the age of diagnosis of depression, and alcohol was the most prevalent substance in this group. All this points to the possibility that the addictive disorder may have relevance in the development of depressive mental pathology, being consistent with research that has documented the relationship between alcohol dependence and the development of depression [4,49].

In relation to the results in HRQoL considering comorbidity and the presence/absence of depressive symptoms, it should be noted that dual depressive patients with symptoms are those who report worse Physical Functioning and, therefore, have more limitations in their daily life, for example with walking, making physical efforts, climbing stairs, and carrying groceries. This observation is similar to that of published data [28] and suggests that depressive symptoms are related to physical limitations in the development of the patient’s daily life. The main clinical implication of this finding is that SUD + MDD patients, who are very frequently encouraged to increase their daily activity levels (using an evidenced-based therapeutic approach), may need more time for their progressive behavioral activation considering their physical limitations. Likewise, when we compare the groups with population values, it is observed that the presence of depressive symptoms is the differential aspect in this quality of life dimension, since dual asymptomatic depressive patients and SUD show scores very similar to those of the normative data. Based on the results of our study (the first one to address the impact of depressive symptoms in HRQoL of dual depression) it is worth noting the need for therapeutic approaches to improve physical functioning, especially for symptomatic patients with SUD + MDD.

On the other hand, in line with previous findings [26], we found that SUD + MDD comorbidity was linked to a worse quality of life as a result of Mental Health, regardless of whether the patient was symptomatic or asymptomatic. The comorbidity between depression and SUD is related to limitations in the patient’s daily life as a result of nervousness, insomnia, and low mood, without the need to experience depressive symptoms at that time. Although the SUD group also reported worse Mental Health compared to population data, it was the SUD + MDD group that showed the greatest limitations in this dimension of HRQoL. Therefore, regardless of the mood of the patient with SUD + MDD at the time the treatment is being carried out, it is a type of patient with whom it would be necessary to use specific techniques aimed at managing anxious and affective symptoms. A clinical implication of this finding is that the SF-36 and its Mental Health dimension seems to be sensitive to the existence of a MDD and it might be used as a screening instrument in addiction’s treatment centers; this observation also adds evidence to previous findings [26,43] about HRQoL as an indicator of mood disorders.

The dual disorder group was the one that showed a lower energy level, more tiredness and exhaustion (Vitality dimension), not being explained by the presence of active symptoms of the MDD. This observation is consistent with previous work [25,29] and, although the SUD group also presented worse Vitality, regarding the normative data, poorer quality of life resulting from a low Vitality was especially linked to SUD + MDD comorbidity. Thus, it may be important to include strategies in treatment programs that help SUD patients to increase their energy levels (such as physical exercise, outdoor activities) [50,51], and this seems especially necessary with SUD + MDD patients. Moreover, as dual depressed patients present insomnia very frequently (according to the Mental Health dimension), they could specially benefit from strategies focused on improving their sleep quality, prioritizing a behavioral approach, but the consideration of a pharmacological treatment is also necessary. All these actions could result in better wakefulness and circadian rhythm adjustment [50]. In addition, the results in the General Health dimension indicated a worse perception by patients with SUD + MDD, not being related to whether they were symptomatic or asymptomatic, as compared to SUD patients and the population mean. Therefore, the existence of the diagnosis of depression comorbid to the SUD is linked to a perception of the patient that they can become ill easily, or that their health is going to worsen, and the emotional affective state of the moment would not be modulating this perception. An approach with cognitive-behavioral techniques in the context of a comprehensive treatment of SUD + MDD patients developing positive thoughts regarding their General Health could be very beneficial. Thus, treatment could be especially focused on cognitive-restructuring and cognitive therapy so as to adjust patients’ perception to their actual mood symptoms and clinical evolution.

Finally, we found that the perception of the Health Change experienced in the last year was higher in patients with SUD and asymptomatic patients SUD + MDD, compared to symptomatic SUD + MDD. This observation is consistent with previous findings that directly related the reduction of depressive symptoms during treatment with a significant improvement in HRQoL [52]. Our data add evidence to the need of integrated treatments models for dual depressed patients addressed to improve patient’s mood and their affective symptoms, instead of treatments that especially aim to achieve abstinence and prevent relapses. Future studies should further explore the specific influence of depressive symptoms on the recovery of the patient with dual disorder. In sum, our results confirm previous observations regarding the complications presented by patients with dual disorders as a diagnostic entity [1,12,14] and provide evidence regarding the need for a global therapeutic approach for patients with dual depression, beyond the mere approach to the typical affective symptoms of MDD.

### Predictive Value of HRQoL Dimensions

Regarding the predictive value of the HRQoL dimensions, we observed that different dimensions are related to different variables at the follow-up points. It seems that the different aspects of HRQoL have a diverse role at prognosis and this role is not so consistent for symptomatic patients SUD + MDD patients. At 3 months of follow-up, we highlight that no dimension was shown to be related to the evolution of symptomatic SUD + MDD patients. However, in asymptomatic MDD patients, we observed that the greater the problems generated by the emotional state (Role Emotional), the greater the probability that the patient was continuing under treatment; in this case, experiencing daily difficulties resulting from emotional state was associated with therapeutic adherence. Future studies may explore if these patients could be experiencing an important emotional support from their therapeutic teams which helps bonding them and keeps them on track with treatment. Relapses at 3 months were related to two different quality of life dimensions depending on whether the patient was asymptomatic SUD + MDD or a patient with SUD. Thus, in patients with SUD, worse physical function (limitations in daily physical activities) was associated with a greater probability of having relapses throughout the year of follow-up (3, 6, and 12 months); these data are in agreement with published studies that associate the use of maladaptive coping strategies with a higher probability of relapse in patients with dual disorders [6,51]. Future studies should deepen this relationship and investigate whether the presence of physical problems in SUD reduces the patient’s motivation to maintain abstinence or whether consumption works as a strategy to cope with these daily limitations. On the other hand, in asymptomatic patients with SUD + MDD, better General Health is associated with a greater presence of relapses. This finding points to the study of the role that the perception of a better general health state plays in the non-compliance with the treatment guidelines and in the possible exposure to risk situations that induce relapses. Therefore, asymptomatic SUD + MDD patients under treatment and better General Health from the SF-36 may be considered as higher risk to relapse patients; emphasizing relapse prevention strategies with them could be especially relevant.

At 6 months of evolution of the patient under treatment for the SUD, it should be noted that a lower presence of Bodily Pain symptoms increased the probability of being discharged from the treatment in those diagnosed with SUD. Thus, it was more probable for patients with SUD and no pain or no limitations due to pain to achieve therapeutic goals significantly so as to be discharged from their treatment center. In contrast, no HRQoL dimension showed predictive value with discharge from treatment in patients with SUD + MDD. The Vitality dimension exhibited different relationships with the evolution of the patient according to his diagnosis. Thus, symptomatic SUD + MDD patients who experienced greater Vitality had a greater probability of continuing treatment at the 6-month follow-up. Future studies should deepen into whether the fact of feeling more energetic and less tired represents a factor in favor of motivation for change or therapeutic adherence. In patients with SUD, greater tiredness and exhaustion (lower vitality) was associated with a greater frequency of needing medical assistance at 6 months of follow-up. Therefore, our data add support to previous works [4,12,13,14] and shows the need of future studies that explore if a higher need of medical services in SUD patients is related to dysphoric feelings, such as tiredness, or whether it is due to other health-associated variables. The improvement of this quality of life dimension in patients with SUD may be a key aspect in order to reduce the patient’s need to require medical care resources different from those already received for the addictive disorder and may promote the reincorporation to the working world.

At 12 months of follow-up, we observed that the lower the limitations caused by their emotional state (greater Role Emotional) in patients with SUD, the greater the probability that they had been discharged from the treatment. In other words, patients with SUD and without problems at work or other daily activities as a result of emotional problems have a good prognosis as they were more likely to achieve treatment goals as to be discharged. Hence, specific interventions to improve the emotional state of the SUD patient could benefit the success of the treatment. On the other hand, in symptomatic patients with SUD + MDD, it was observed that having a higher energy level (greater Vitality) increased the probability that they were still receiving treatment for SUD at one year of follow-up, while the experience of limitations as a result of physical pain (minor Bodily Pain) increased the probability of treatment discontinuation (drop-out). In the latter case, future research could assess the influence of physical pain on the motivation of the symptomatic patient with SUD + MDD to stay adhered to the treatment for SUD. Finally, we also observed at one year of follow-up in asymptomatic patients with SUD + MDD that the probability of relapse increased as the state of General Health was better, while in SUD patients we again found that the greater the physical limitations in daily functioning (Physical Functioning) the higher the probability of relapse. In this way, it is observed that the different dimensions in HRQoL point to possible lines of therapeutic intervention in patients with SUD and, taking into account the psychiatric comorbidity with MDD, they contribute aspects of great clinical interest for the future based on the presence/absence of depressive symptoms.

The present study has several strengths and some possible limitations. We highlight as a strong point that this is the first work that analyzes HRQoL in a sample of patients with SUD + MDD, evaluating their symptomatic or asymptomatic depressive state, and comparing them with a group of patients with only SUD diagnosis, as well as with population data. In addition, other strong points of our study are that we have explored the predictive role of the different dimensions of HRQoL at 3, 6, and 12 months of follow-ups and provided data with clinical utility for the management of the patient under treatment for SUD. As possible limitations, we can point out that our sample is made up of only men, thus limiting the generalization of results to male patients in treatment for SUD. The data on the dimensions of HRQoL come from a self-reported questionnaire, thus being subjective data resulting from the individual perception of each patient. Future studies should overcome these limitations and confirm our findings with complementary objective measurements that increase knowledge at both theoretical and applied levels.

## 5. Conclusions

Patients with SUD + MDD comorbidity show a worse HRQoL as compared to patients with SUD and existing population data. In the Mental Health, Vitality, and General Health dimensions, the worst quality of life is observed regardless of whether the patient with SUD + MDD is symptomatic or asymptomatic, while in the Physical Functioning dimension, as well as in the Health Change dimension, it is the presence of depressive symptoms that seems to explain the worse quality of life and not the psychiatric comorbidity. The analysis of the HRQoL dimensions must consider whether the patient with SUD + MDD is symptomatic/asymptomatic for a better interpretation of the results and their integration in the treatment.

In relation to the possible influence of HRQoL on the evolution of the patient at 3, 6, and 12 months of follow-up, a differential analysis is also essential, considering the depressive psychiatric comorbidity and the symptomatic/asymptomatic affective state of the patient. Thus, we did not observe relapse predictors in patients with symptomatic SUD + MDD in any of the follow-ups, while we found predictive value in all measurements throughout the year of follow-up (3, 6, and 12 months) for relapses, in General Health for asymptomatic patients with SUD + MDD and in Physical Functioning for SUD. The different dimensions of the SF-36 seem to have a greater predictive value for asymptomatic SUD + MDD and SUD patients than for symptomatic SUD + MDD patients. Future studies should deepen this line of research, as well as assess the influence of specific interventions aimed at improving the different dimensions of HRQoL in patients under treatment for SUD, with and without depressive comorbidity.

## Figures and Tables

**Table 1:** Sociodemographic and clinical data for the two groups. Means and standard deviation or percentages, and statistical contrasts.

	SUD + MDD (N = 114)	SUD (N = 123)	Statistical Contrast
**Sociodemographic Data**			
Age (years)	41.55 ± 8.64	37.32 ± 8.27	*t*_(235)_ = 3.83 ***
Marital status			*χ*^2^_(4)_ = 9.77
Single	46.4%	56.6%	
Stable partner	6.3%	7.4%	
Married	8.9%	13.9%	
Separated/divorced	35.7%	22.2%	
Widower	2.7%	0%	
Years of schooling	11.17 ± 2.84	10.75 ± 2.43	*t*_(235)_ = 1.23
Economic situation			*χ*^2^_(4)_ = 30.02 ***
Active	10.7%	28.7%	
Unemployed	25.9%	28.7%	
Disability pension	44.6%	14.8%	
Sick leave (due SUD treatment)	8.9%	10.7%	
No income	9.8%	17.2%	
**Medical and Psychiatric Data**			
Medical disease comorbidity ^a^	50%	31%	*χ*^2^_(6)_ = 14.98 **
Hypercholesterolemia	9.5%	2.4%	
Respiratory system disease	10.7%	8.2%	
Hepatitis	8.0%	7.4%	
Diabetes	3.6%	1.6%	
Hypertension	6.3%	2.5%	
HIV	4.5%	5.7%	
Other	2.5%	6.3%	
Daily number of medications	2.94 ± 1.63	0.83 ± 1.27	*t*_(235)_ = 10.97 ***
Type of medication prescribed ^a^			
Antidepressants	71.0%	18.9%	*χ*^2^_(1)_ = 63.93 ***
Anxiolytics	42.4%	9.8%	*χ*^2^_(1)_ = 33.16 ***
Mood Stabilizers	42.6%	13.9%	*χ*^2^_(1)_ = 25.96 ***
Disulfiram	26.2%	15.2%	*χ*^2^_(1)_ = 4.45
Other	28.7%	13.9%	*χ*^2^_(1)_ = 25.78 ***
History of suicide attempt	46.4%	19.0%	*χ*^2^_(1)_=21.72 **
Number of lifetime suicidal attempts	1.06 ± 2.08	0.32 ± 0.77	*t*_(235)_ = 3.65 ***
**SUD-Related Data**			
Quantity of substance used ^a^	2.69 ± 1.48	2.56 ± 1.64	*t*_(235)_ = 0.66
Substance of use ^a^			
Alcohol	83.0%	72.1%	*χ*^2^_(1)_ = 3.96
Cocaine	76.8%	86.9%	*χ*^2^_(1)_ = 5.27
Cannabis	42.9%	39.3%	*χ*^2^_(1)_ = 0.98
Hallucinogens	19.6%	18.9%	*χ*^2^_(1)_ = 0.23
Opioids	18.8%	16.4%	*χ*^2^_(1)_ = 0.22
Sedatives	14.3%	6.6%	*χ*^2^_(1)_ = 3.78
DAST-20	13.10 ± 4.40	12.73 ± 3.78	*t_(_*_235)_ = 0.65
Main substance of dependence			*χ*^2^_(4)_ = 7.88
Alcohol	12.5%	10.4%	
Cocaine	11.5%	12.4%	
Alcohol and cocaine	34.9%	34.4%	
Alcohol and sedatives	2.7%	1.8%	
Polydrug use	38.4%	41.0%	
Mean abstinence period (months)	9.95 ± 5.65	7.52 ± 2.93	*t*_(235)_ = 1.40
Substance use disorder age onset (years)	21.56 ± 9.71	20.63 ± 7.70	*t*_(235)_ = 0.81
Years of substance use disorder	19.10 ± 10.69	15.93 ±9.32	*t*_(235)_ = 2.44

SUD + MDD: Substance use disorder with comorbid depression; SUD: Substance use disorder; DAST-20: Drug Abuse Screening Test; HDRS: Hamilton Depression Rating Scale; ^a^ Percentages will not equal 100 as each participant may be in more than one category at the same time; ** *p* < 0.01; *** *p* ≤ 0.001.

**Table 2:** Clinical measures (means and standard deviation) for de dual disorder group with Substance Use Disorder and Major Depressive Disorder according to the presence/absence of depressive symptoms.

	Symptomatic SUD + MDD (N = 51)	Asymptomatic SUD + MDD (N = 63)	Statistical Contrast
Major depressive disorder age onset (years)	28.65 ± 9.35	30.16 ±8.78	*t* = 0.865
Years of major depressive disorder	13.96 ±9.46	10.50 ± 7.93	*t* = −2.60
Hamilton Depression Rating Scale	12.39 ± 4.78	4.03 ± 3.08	*t* = −7.48 ***
Social Adaption Self-evaluation Scale	34.80 ± 8.21	38.43 ± 7.51	*t* = −1.45

SUD + MDD: Substance use disorder with comorbid depression. *** *p* < 0.001.

**Table 3:** Health-related quality of life results according to the diagnosis. First multivariate analyses of covariance (MANCOVA) analysis with descriptive statistics (mean and standard error), normative data, F, and eta square (*ηp*^2^) tests.

SF-36 Dimensions	SUD + MDD (N = 114)	SUD (N = 123)	*F*_(2,235)_	ηp^2^
Physical Functioning	88.34 ± 1.35	92.36 ± 1.30	4.443 *	0.019
Social Functioning	70.50 ± 2.50	78.65 ± 2.40	5.334 *	0.022
Role Physical	77.42 ± 3.25	77.20 ± 3.12	0.002	0.001
Role Emotional	53.07 ± 3.72	66.39 ± 3.57	6.471 *	0.027
Mental Health	52.29 ± 1.59	65.65 ± 1.53	35.458 ***	0.133
Vitality	50.36 ± 1.73	66.61 ± 1.66	44.550 ***	0.161
Bodily Pain	72.34 ± 2.53	76.68 ± 2.44	1.479	0.006
General Health	62.84 ± 1.75	73.42 ± 1.68	18.436 ***	0.074
Health Change item	77.50 ± 2.21	90.64 ± 2.12	17.849 ***	0.071
Physical Composite Scale	60.19 ± 1.54	61.39 ± 1.48	0.305	0.001
Mental Composite Scale	42.71 ± 1.63	51.60 ± 1.56	14.961 ***	0.061

SF-36: The Short Form Health Survey; SUD + MDD: Substance Use Disorder with comorbid Major Depression Disorder; SUD: Substance Use Disorder; * *p* < 0.05; *** *p* ≤ 0.001.

**Table 4:** Health-related quality of life results according to the diagnosis and presence/absence of depressive symptoms. Second MANCOA analysis with descriptive statistics (mean and standard error), normative data, F, and eta square (*ηp*^2^) tests.

SF-36 Dimensions	SUD + MDD Symptomatic (N = 51)	SUD + MDD Asymptomatic (N = 63)	SUD (N = 123)	F	ηp^2^	Contrasts
Physical Functioning	83.42 ± 1.98	92.02 ± 1.77	92.36 ± 1.30	7.230 ***	0.059	SUD and SUD + MDD asymptomatic > SUD + MDD symptomatic
Social Functioning	70.41 ± 3.74	70.58 ± 3.35	78.65 ± 2.40	2.656	0.022	
Role Physical	71.60 ± 4.81	82.07 ± 4.31	77.20 ± 3.12	1.331	0.011	
Role Emotional	52.18 ± 5.55	53.78 ± 4.97	66.39 ± 3.57	3.246	0.027	
Mental Health	49.03 ± 2.36	54.90 ± 2.11	65.65 ± 1.53	19.662 ***	0.145	SUD > SUD + MDD symptomatic/asymptomatic
Vitality	48.42 ± 2.57	51.91 ± 2.30	66.61 ± 1.66	22.800 ***	0.165	SUD > SUD + MDD symptomatic/asymptomatic
Bodily Pain	68.63 ± 3.77	75.30 ± 3.37	76.68 ± 2.44	1.625	0.014	
General Health	62.86 ± 2.61	62.83 ± 2.34	73.42 ± 1.68	9.178 ***	0.074	SUD > SUD + MDD symptomatic/asymptomatic
Health Change item	83.78 ± 3.25	72.49 ± 2.91	77.50 ± 2.21	12.547 ***	0.098	SUD and SUD + MDD asymptomatic > SUD + MDD symptomatic
Physical Composite Scale	59.37 ± 2.30	60.84 ± 2.06	61.40 ± 1.48	0.26	0.002	
Mental Composite Scale	42.77 ± 2.43	42.67 ± 2.18	51.60 ± 1.57	7.449 ***	0.176	SUD > SUD + MDD symptomatic/asymptomatic

SF-36: The Short Form Health Survey; SUD + MDD: Substance Use Disorder with comorbid Major Depression Disorder; SUD: Substance Use Disorder; *** *p* ≤ 0.001.

**Table 5:** Results from the logistic and linear regression models for health-related quality of life dimensions and follow-up data at 3, 6, and 12 months for the groups.

SF-36 Dimensions	Follow-Up Data Variables at 3 Months	SUD + MDD Symptomatic (N = 51)	SUD + MDD Asymptomatic (N = 63)	SUD (N = 123)
Role Emotional	Being at treatment		*OR* = 0.979 *	
			*β* = −0.022	
Physical Functioning	Relapses			*OR* = 0.957 **
				*β* = −0.044
General Health	Relapses		*OR* = 1.028 *	
			*β* = 0.028	
Vitality	Quantity of medical consultations		*R*^2^ = 0.261 ***	
			*β* = −0.527	
Vitality	Need for medical assistance			*OR* = 0.949 *
				*β* = −0.052
	**Follow-up Data Variables at 6 Months**	**SUD + MDD Symptomatic** **(N = 51)**	**SUD + MDD Asymptomatic** **(N = 63)**	**SUD** **(N = 123)**
Bodily Pain	Discharge from treatment			*OR* = 1.023 *
				*β* = 0.022
Vitality	Being in treatment	*OR* = 1.046 *		
		*β* = 0.045		
Physical Functioning	Relapses			*OR* = 0.965 *
				*β* = −0.036
General Health	Quantity of medical consultations		*R*^2^ = 0.304 ***	
			*β* = −0.570	
Vitality	Need for medical assistance			*OR* = 0.915 **
				*β* = −0.089
	**Follow-up Data Variables at 12 Months**	**SUD + MDD Symptomatic** **(N = 40)**	**SUD + MDD Asymptomatic** **(N = 45)**	**SUD** **(N = 87)**
Role Emotional	Discharge from treatment			*OR* = 1.014 *
				*β* = 0.014
Vitality	Being in treatment	*OR* = 1.064 *		
		*β* = 0.062		
Bodily Pain	Drop-out treatment	*OR* = 0.959 **		
		*β* = −0.042		
Physical Functioning	Relapses			*OR* = 0.958 *
				*β* = −0.042
General Health	Relapses		*OR* = 1.035**	
			*β* = 0.035	
General Health	Quantity of medical consultations		*R*^2^ = 0.262**	
			*β* = −0.535	

SF-36: The Short Form Health Survey; SUD + MDD: Substance Use Disorder with comorbid Major Depression Disorder; SUD: Substance Use Disorder; * *p* ≤ 0.05; ** *p* < 0.01; *** *p* < 0.001.

**Table 6:** Attrition analysis for sociodemographic and clinical variables between patients who had/did not had follow-up data at 12-months. Means and standard deviation or percentages, and statistical contrasts.

	With 12-Months Follow-Up Baseline Data (N = 172)	Without 12-Months Follow-Up Baseline Data (N = 65)	Statistical Contrasts
**Sociodemographic Data**			
Age (years)	39.55 ± 8.69	38.00 ± 8.74	*t*_(235)_ = 0.91
Marital status			*χ*^2^_(4)_ = 8.44
Single	57%	56%	
Stable partner	7.4%	6.3%	
Married	12.3%	13.7%	
Separated/divorced	21.8%	24.0%	
Widower	1.5%	0%	
Years of schooling	10.92 ± 2.59	11.13 ± 2.95	*t*_(235)_ = − 0.41
Economic situation			*χ*^2^_(4)_ = 6.69
Active	21.6%	23.3%	
Unemployed	25.0%	27.0%	
Disability pension	29.4%	26.7%	
Sick leave (due SUD treatment)	9.3%	13.3	
No income	14.7%	9.7%	
**Medical and Psychiatric Data**			
Medical disease comorbidity ^a^	33.3%	30.1%	*χ*^2^_(6)_ = 6.18
Hypercholesterolemia	3.4%	2.3%	
Respiratory system disease	10.5%	8.8%	
Hepatitis	8.4%	10.0%	
Diabetes	2.9%	1.9%	
Hypertension	4.9%	6.3%	
HIV	4.9%	6.7%	
Other	11.3%	14.2%	
Daily number of medications	1.90 ± 1.84	1.21 ± 1.29	*t*_(235)_ = 10.97 ***
Type of medication prescribed ^a^			
Antidepressants	43.7%	39.3%	*χ*^2^_(1)_ = 1.78
Anxiolytics	26.6%	23.9%	*χ*^2^_(1)_ =1.26
Mood Stabilizers	12.9%	10.7%	*χ*^2^_(1)_ = 5.66
Disulfiram	10.2%	12.5%	*χ*^2^_(1)_ = 0.99
Other	13.5%	14.8%	*χ*^2^_(1)_ = 1.69
History of suicide attempt	32.0%	33.3%	*χ*^2^_(1)_ =2.91
Number of lifetime suicidal attempts	0.68 ± 1.65	0.63 ± 1.00	*t*_(235)_ = 0.165
**SUD Related Data**			
Quantity of substance used ^a^	2.58 ± 1.55	2.90 ± 1.78	*t*_(235)_ = − 1.04
Substance of use ^a^			
Alcohol	80.3%	83.7%	*χ*^2^_(1)_ = 3.14
Cocaine	82.4%	80%	*χ*^2^_(1)_ = 5.98
Cannabis	40.1%	43.3%	*χ*^2^_(1)_ = 0.76
Hallucinogens	19.6%	16.7%	*χ*^2^_(1)_ = 0.14
Opioids	17.9%	18.1%	*χ*^2^_(1)_ = 0.41
Sedatives	5.6%	8.3%	*χ*^2^_(1)_ = 4.39
DAST-20	13.59 ± 4.16	14.48 ± 3.43	*t_(_*_235)_ = 1.20
Main substance of dependence			
Alcohol	10.3%	8.7%	
Cocaine	10.8%	9.3%	
Alcohol and cocaine	29.7%	33.3%	
Alcohol and sedatives	1.5%	2.3%	
Polydrug use	39.7%	40.0%	
			
Mean abstinence period (months)	8.67 ± 4.41	7.20 ± 4.03	*t*_(235)_ = 0.68
Substance use disorder age onset (years)	21.45 ± 9.02	18.53 ± 6.08	*t*_(235)_ = 1.71
Years of substance use disorder	17.34 ± 10.16	18.26 ± 9.93	*t*_(235)_ = − 0.47

SUD + MDD: Substance use disorder with comorbid depression; SUD: Substance use disorder; DAST-20: Drug Screening Test; HDRS: Hamilton Depression Rating Scale; ^a^ Percentages will not equal 100 as each participant may be in more than one category at the same time.

**Table 7:** Clinical measures (means and standard deviation) for de dual disorder group with Substance Use Disorder and Major Depressive Disorder with/without follow-up data at 12 months.

	SUD + MDD with 12-Months Follow-Up Baseline Data (N = 85)	SUD + MDD without 12-Months Follow-Up Baseline Data (N = 29)	Statistical Contrasts
Major depressive disorder age onset (years)	29.77 ± 9.23	27.71 ± 7.99	*t* = 0.86
Years of major depressive disorder	12.39 ±8.70	10.82 ± 9.59	*t* = 0.69
Hamilton Depression Rating Scale	9.72 ± 5.80	10.50 ± 5.82	*t* = − 0.47
Social Adaption Self-evaluation Scale	35.56 ± 7.19	36.30 ± 10.87	*t* = − 0.286

SUD + MDD: Substance use disorder with comorbid depression.
